# Globalisation in the time of COVID-19: repositioning Africa to meet the immediate and remote challenges

**DOI:** 10.1186/s12992-020-00581-4

**Published:** 2020-06-24

**Authors:** Sanni Yaya, Akaninyene Otu, Ronald Labonté

**Affiliations:** 1 School of International Development and Global Studies, Faculty of Social Sciences, University of Ottawa, 120 University Private, Ottawa, ON K1N 6N5 Canada; 2 The George Institute for Global Health, University of Oxford, Oxford, UK; 3 Department of Infection and Travel Medicine, Leeds Teaching Hospitals NHS Trust, Leeds, UK; 4 Department of Internal Medicine, College of Medical Sciences, University of Calabar, Calabar, Cross Rivers State Nigeria; 5 School of Epidemiology and Public Health, Faculty of Medicine, University of Ottawa, 600 Peter Morand Crescent, Ottawa, Ontario K1G 5Z3 Canada

**Keywords:** COVID-19, Globalisation, Governance, Economic policies, Recession, Global health, Africa

## Abstract

The COVID-19 pandemic has ushered in a new climate of uncertainty which is fuelling protectionism and playing into nationalist narratives. Globalisation is under significant threat as governments scramble to reduce their vulnerability to the virus by limiting global trade and flows of people. With the imposition of border closures and strict migration measures, there have been major disruptions in Africa’s global supply chains with adverse impacts on employment and poverty. The African economies overly reliant on single export-orientated industries, such as oil and gas, are expected to be severely hit. This situation is further aggravated by tumbling oil prices and a lowered global demand for African non-oil products. The agricultural sector, which should buffer these shocks, is also being affected by the enforcement of lockdowns which threaten people’s livelihoods and food security. Lockdowns may not be the answer in Africa and the issue of public health pandemic response will need to be addressed by enacting context-specific policies which should be implemented in a humane way. In addressing the socioeconomic impact of COVID-19 on African nations, we argue that governments should prioritize social protection programmes to provide people with resources to maintain economic productivity while limiting job losses. International funders are committing assistance to Africa for this purpose, but generally as loans (adding to debt burdens) rather than as grants. G20 agreement so suspend debt payments for a year will help, but is insufficient to fiscal need. Maintaining cross-border trade and cooperation to continue generating public revenues is desirable. New strategies for diversifying African economies and limiting their dependence on external funding by promoting trade with a more regionalised (continental) focus as promoted by the African Continental Free Trade Agreement, while not without limitations, should be explored. While it is premature to judge the final economic and death toll of COVID-19, African leaders’ response to the pandemic, and the support they receive from wealthier nations, will determine its eventual outcomes.

## Background

Even before COVID-19, globalisation was already under significant threat from rising nationalism forcing governments and businesses to define new constructs and priorities [1]. This response to the conflicting forces of globalisation and nationalism has given rise to the term “slowbalisation”, coined by *The Economist* to describe declines in trade, multinational profits, and foreign investment and leading to arguments that we have now passed ‘peak globalisation’. The COVID-19 pandemic appears to have introduced additional fear and uncertainty among populations, resulting in new behaviours and beliefs [2]. People are becoming more suspicious and less accepting of foreign things and all this is occurring on a background of increasing anarchy in global governance [2]. The economic interdependence and multilateral norms or rules that globalisation emphasised over the past several decades, creating the global supply chains that contributed to the economic growth many low- and middle-income countries experienced since the 1990s, is facing formidable and existential threats. Although arguments that the globalisation era is over, or at least on the wane, may be premature, the economic impacts of the pandemic are rattling its inherent assumptions with little clear indication of what may follow in its (eventually) subsiding wake. In the meanwhile, the economic interdependence that characterized recent globalisation is becoming unglued, and with it the policy assumptions of many governments worldwide and the (already) fragile livelihoods of billions of people. Nowhere is the concern over the health impacts of this greater than in Africa.

Whilst governments across Africa hasten to reinforce measures to contain the spread of COVID-19 in the context of fragile health systems, several pertinent questions arise.^1^ How can socio-economic development stimulated by globalisation practices related to increased international trade be sustained or, if not, reformed in ways that still enhance livelihood opportunities? How might African governments successfully limit community transmission of COVID-19 while also providing economic relief to families and businesses affected by physical distancing or ‘lockdown’ strategies? In this commentary we explore the challenges facing most African countries in improving the population health outcomes in the current COVID-19 and future pandemics, while promoting a renewed globalisation based upon health and social development goals and not solely on economic growth measures.

## Socioeconomic impacts of COVID-19 – can globalisation prevail in the face of the impeding recession?

As the COVID-19 crisis continues to deepen, the significant health and economic consequences of this disease is crippling even the most developed nations. With < 0.5% of globally confirmed cases occurring in Africa, the continent appears so far to be relatively spared the untoward direct health consequences of the COVID-19 pandemic [3]. The disease nonetheless has already had a destabilizing effect on the lives of millions of Africans with disproportionate impact on the poor and underserved [4]. The interconnectedness that characterises globalisation has brought economic benefits to many African countries. With COVID-19, there have been disruptions in Africa’s global supply chains in the face of tumbling oil prices and a lowered global demand for African non-oil products, which constitutes a threat to the economic stability of the continent. Projected losses from oil shocks alone may result in a reduction in Africa’s export revenues by about US$101 billion in 2020 [5]. This dip in oil prices will disproportionately put in economic and fiscal peril resource-dependent countries like Angola, the Democratic Republic of the Congo (DRC), Nigeria as well as other oil importing African countries. .

Before the virus spread into Africa, the International Monetary Fund (IMF) in mid-February 2020 warned the continent of the impending risk of an economic slowdown as China, where the virus emerged, is the largest trading partner and foreign investor of many countries in the continent [6]. Many African countries are interconnected to affected economies of the United States and European Union. The growth deceleration in these major economies will have a negative impact on price of goods exported from Africa such as mineral ores and metals [7]. Conservative estimates suggest that COVID-19 could cause Africa’s GDP to drop by as much as three to eight percentage points with projected economic losses of between US$90 and US$200 billion in 2020 alone [4]. The United Nations Development Programme (UNDP) estimate that this pandemic could result in the loss of nearly half of all jobs in Africa where unemployment is already a major concern [8]. This is likely to further aggravate Africa’s fragile economic situation, one in which as many as 422 million people (one in three Africans) are estimated to be living below the international poverty line, i.e. $1.90 per day [8].

In an attempt to fight COVID-19, many African countries have adopted some international policy trends such as border closures, strict migration measures, imposition of quarantines, and enforcement of stay-at-home orders [9]. These measures embody the dialectical quality of contemporary globalisation [10]: on the one hand they reflect the rapid communicative and even hegemonic nature of global knowledge exchange; while on the other hand they accentuate boundaries instead of eroding them and limit interactions across socio-economic, political, and technological spheres. This substantial disruption in globalisation’s economic integration has led to retardation of key sectors such as air transportation and tourism, with a concomitant reduction in trade, remittances, and investments. In the face of waning official development assistance to the continent and capital flight, unemployment and food insecurity is likely to be exacerbated across the continent [5].

## The role of economic policies and collaborative efforts in mitigating the effect of COVID-19

In dealing with the pandemic and its socioeconomic impact on African nations, it is vital that interventions focus on women, children, individuals with disabilities, youth, older people, low-income workers, and small and medium enterprises. These and other vulnerable groups, such as those working in the informal sector, internally displaced individuals, and refugees, are more likely to suffer the devastating social and economic consequences of the virus [11, 12]. These socioeconomic consequences could precipitate health problems such as depression, anxiety, post-traumatic stress disorder, suicidality, child abuse and intimate-partner violence among these vulnerable groups [13, 14].

Providing loans and credit guarantees with limited conditionality can be one means to invigorate private sector participation in continuing economic productivity, increase the liquidity of small-scale businesses, and limit job losses. Such financial assistance may have to be provided directly by African governments by utilizing innovative strategies. Groups such as the World Bank, International Monetary Fund (IMF) and the European Investment Bank are making funds available to Africa, but primarily as loans. This will only increase the debt burdens already being carried by most of these countries. Already, many African countries have risky debt profiles as 24 of the 47 countries have current account deficits that exceed 5% of their GDP, with the external debts of six countries exceeding 40% of their GDP [15]. The existing weak fiscal capacity of African countries, however, could be bolstered by providing international or multilateral financial assistance as grants, rather than as loans; and by suspending or cancelling much of their current debt owed to relevant financial bodies (such as development banks, the IMF, and bilateral donors). The G20 countries in April did agree to suspend debt payments for poor countries (most of which are in Africa), which will free up US$20 billion for government COVID-19 interventions [16]. This one-year suspension will provide some fiscal space for governments to focus on crafting economic relief packages with limited (if any) policy conditionalities (notably renewed austerity) that would otherwise be likely to constrain future health and social protection spending [17]. But the amount is considered woefully inadequate relative to need, and should be extended to all forms of multilateral and privately held debt.

The current pandemic crisis also presents an opportunity to explore new strategies for diversifying African economies and limiting their dependence on external funding (whether loans, grants, or investments) by promoting trade and a more regionalised (continental) form of globalisation. The African Continental Free Trade Agreement (AfCFTA) (among 54 of the 55 African Union nations, with only Eritrea so far now yet signing the agreement) which was due to commence in July 2020 may hold some promise in this regard. The driving principle behind AfCFTA is to eliminate tariffs over a five to eight-year period for 90% of goods, with eventually all tariffs on all goods removed, to promote trade in goods and services between African countries [18]. This agreement could be activated and, although its economic benefits are being overstated [19], stimulus packages could be provided to facilitate cross-border trade in the continent. There are critiques of the AfCFTA agreement, that not all countries within the same continent are equal (indeed, just three countries – Nigeria, South Africa, and Egypt – account for over 50% of the continental GDP) [20], and the larger, wealthier ones could quickly come to dominate continental markets without compensatory social agreement. Past history of trade agreements further suggests that benefits generally accrue to already economically elite groups, and Africa is already one of the world’s most economically unequal regions. Specific policies will need to be built-in to circumvent such unfavourable outcomes.

Maintaining cross-border trade and cooperation could potentially maintain some financial resources for assist many high-risk African countries to fight the pandemic, although an overhaul of trade agreements to ensure equitable gains and ecological sustainability is more likely to be a post-pandemic undertaking. In the nearer term, African countries could unite in collectively reducing their tariffs on all medical supplies related to COVID-19 and regulating their domestic prices. Under African Union and/or the World Health Organization (WHO) Regional Office for Africa auspices, member states could enter into cost-sharing agreements to ensure that larger African countries with deeper fiscal pockets (or borrowing capacities) do not outbid smaller, poorer nations. Whether such safeguard measures are entered into new AfCFTA rules, or become part of African-wide demands in new, or amended, agreements with countries beyond the continental borders, is moot; but trade agreements and increased trade do not necessarily overcome inequities in wealth and power.

## Politics and governance in the wake of COVID-19 – where should the focus of Africa leaders lie?

The COVID-19 pandemic poses a daunting challenge to African leaders and it is imperative that they work very closely with scientists, policy experts, and medical specialists to design feasible plans. High-level political advocacy combined with multi-sectoral and multi-national globalisation efforts such as the Africa Taskforce for Coronavirus Preparedness and Response (AFTCOR) are vital. An example of this is the US$3 billion Fight COVID-19 Social Bond provided by the African Development Bank to alleviate the social and economic impact of the pandemic on African countries. The U.S. Agency for International Development (USAID) announced a commitment of US$37 million in March 2020, to finance 25 affected or high-priority countries including Angola, South Africa, Zambia, Zimbabwe, Ethiopia, Nigeria; this is to help prepare laboratories for large scale testing, investigate and implement contact tracing, train health workers, and implement public health emergency plans, among other policy actions [21–23].

Short-term fiscal policy redirecting government expenditure to back economic relief to families and businesses will need to be drafted and deployed. Wage subsidies, credit guarantees, and postponed financial obligations like loan repayments will be of immense benefit especially for the vulnerable segments of the population [24]. Although African governments generally lack the same options to create new money (via quantitative easing via government bond purchases by central banks) that high income countries (HICs) have, South Africa being one exception [25], innovative ways of providing this relief from available limited resources will need to be sought. Economic development plans could be revisited with a view to reallocation of the existing planned budget to tackle COVID-19 [26]. This reflects a more sustainable approach as even some high-income countries are beginning to re-impose fiscal austerity, or to broach the idea of soon doing so, to begin reducing the scale of publicly assumed debt [27]. In all this, ensuring transparency and accountability is of paramount importance for securing buy-in from the populace. In Nigeria, for instance, the government failed to provide vital details for a cash transfer program implemented to cushion household expenditure, and this raised questions and doubts about the criteria for selecting beneficiaries; many sensed political influences in the decision-making process leading to a crisis of confidence in the leaders [9].

## How do socio-economic indices and healthcare in Africa intersect in COVID-19?

The efficacy of any country’s response to the COVID-19 pandemic is largely dependent on the competence of its healthcare systems. The fragile nature of Africa’s health systems has been associated with perennial inadequacies in public health spending and critical shortages of health professionals. Statistics from Africa suggest that there is just one doctor and seven hospital beds per 1000 persons, 1.06 midwives and nurses for every 1000 persons and less than 50% of the population have access to modern healthcare services [28, 29]. Against the backdrop of such abysmal health indices, specific policies are urgently required to bolster capacity for surveillance, mass testing, management of severe cases of COVID-19, health-worker training, supply-chain management and community engagement [28]. An estimated amount of US$10.6 billion increase in health spending will be needed to curb the spread of the virus in Africa [30]. This means that funds will have to be rerouted from other development activities to focus on this unanticipated and unplanned health spending. Such moves are likely to impair economic growth, unless new sources of public revenue are quickly developed including, as examples, new forms of capital control (to avoid capital flight), closure of offshore financial centres (‘tax havens’), and more extensive, progressive, and transparent systems of national taxation. Even so, the need for international transfers will still be required, as most studies suggest that Africa will be unable to self-finance its development goals (i.e. the Agenda 2030 goals) without ongoing development assistance [31]. As one example, a study of thirteen African countries facing high burdens of HIV, TB, and malaria found that, on average, they relied upon international financing for 84, 64, and 64%, respectively, of the disease programme costs over the 2015–2017 period [32].

## Questioning African adoption of strict lockdowns and physical distancing measures to control COVID-19

In China, the government’s approach to COVID-19 of strict imposition of quarantine, deployment of mass testing and meticulous contact tracing appears to have had dividends, although this came at the human and economic cost [33]. However, Africa’s approach to the imposition of measures aimed at achieving physical distancing in COVID-19 has varied widely across the continent. In Tanzania, Burundi and Zimbabwe, governments and officials have viewed the virus as a punishment and a disease of Western countries. The president of Tanzania, for example, failed to close churches based on the claim that the virus, being satanic, cannot advance in churches [34]. The lockdown of fresh food markets in many African countries have changed the eating habits and diets of many individuals. School closures are estimated to have impacted more than 87% of the world school population; this has resulted in children missing out on school meals which were the only source of nutrition for those from vulnerable households [12, 35]. Excessive lockdown measures could also limit access to vital medical supplies and other necessities of life and impact on people’s welfare.

There are arguments that self-quarantining and physical distancing measures will be literally impossible to achieve in Africa given that up to 70% of city dwellers live in overcrowded slums [15]. Consequently, careful consideration will be needed to establish policies backing lock-downs and physical distancing strategies. These measures are not intended to stop the pandemic, but slow down community transmission of COVID-19 and prevent overwhelming intensive care units (ICUs) where severe cases are managed. Given that COVID mortality in HICs is highest in elderly populations with pre-disposing medical conditions, and that this population is comparatively smaller in Africa than elsewhere; and that ICU capacity is severely limited in Africa even pre-COVID-19, there is a reasonable risk that lock-downs that plunge large populations into poverty and hunger will do more health harm than good. It is estimated that farmers constitute more than 60% of populations in Sub Saharan Africa and agricultural activities account for about 23% of the GDP in the region [26]. Imposing lock-downs in such settings is likely to result in a decline in GDP and to constitute a threat to food security with attendant consequences. Indeed, lockdown measures in response to COVID-19 could double the number of the world’s population facing imminent starvation by the end of 2020, from 135 million to 270 million [36].

The issue of public health pandemic response in Africa will need to be addressed by enacting context-specific policies which should be implemented in a humane way. In countries like Kenya, DRC, Uganda and South Africa, security forces have used excessive force to implement lockdown and stay-at-home orders which has resulted in deaths of civilians [9]. While enforcing these measures can limit the spread of COVID-19, the forceful actions violate human rights to dignity, equal treatment, and life. Governments and political leaders should not use the pandemic as an excuse to constrain individual freedom but as an opportunity to increase trust in government institutions as they manage this public health crisis [9, 29]. Rather, and since part of the health-negative socioeconomic fallout of the pandemic is a result of lockdown measures in high-income countries, it is not unreasonable for African governments to seek specific assistance (as grants, not loans) to compensate for some of the economic costs of their lockdown measures.

## African public health management now in the global spotlight

The public health management capability for containing outbreaks in Africa has certainly improved over the last decade following experiences of dealing with outbreaks such as Ebola, Lassa fever, meningitis, cholera and measles [22]. COVID-19, however, presents a more profound danger as asymptomatic individuals can infect others [29]. The most vulnerable in this crisis are those living in densely populated areas or underserved areas, working in the precarious informal economy, and simply those who are the poorest [12]. Such individuals form a large proportion of Africa’s population. With the lack of potable water and sanitation in rural areas and the existence of urban slums (informal settlements) in many African countries, frequent handwashing as a basic and effective preventive measure against COVID-19 will be unattainable, increasing the risk of infection within and across countries [12].

Whilst African countries have marshalled a robust response to COVID-19 by implementing screening exercises for suspected cases at entry ports and public places, the actual levels of testing have remained low. Unsurprisingly, so has the number of cases and deaths, relative to other continents [37], although Africa’s numbers are expected to rise daily [38].

## Conclusion

The importance of a global health focus on what remains the poorest and most resource-constrained region of the world cannot be overstated, just as the capacities and historic strengths of African societies cannot be understated. How well and how equitably African leaders within their own means respond to the present pandemic, and how well and respectfully they are assisted in doing so by leaders of the world’s wealthier nations, remains the litmus-test of our collective ability to strengthen global health security for all.

## Data Availability

Not applicable.

## Footnotes

1 With some hesitation, we refer to Africa in a continental sense, with only the occasional differentiation by specific African countries. We recognize the enormous diversities within the continent, ethnically, geographically, economically, environmentally, culturally. But there remain COVID-19 challenges that, in a globalising context, are usefully considered at a larger geopolitical scale.
